# Acquired and Innate Immunity Impairment and Severe Disseminated *Mycobacterium genavense* Infection in a Patient With a NF-κB1 Deficiency

**DOI:** 10.3389/fimmu.2018.03148

**Published:** 2019-01-29

**Authors:** Luis Ignacio Gonzalez-Granado, Raquel Ruiz-García, Javier Blas-Espada, José Manuel Moreno-Villares, Marta Germán-Diaz, Marta López-Nevado, Estela Paz-Artal, Oscar Toldos, Yolanda Rodriguez-Gil, Jaime de Inocencio, Nerea Domínguez-Pinilla, Luis M. Allende

**Affiliations:** ^1^Primary Immunodeficiencies Unit, Department of Pediatrics, School of Medicine, University Hospital 12 de Octubre, Complutense University of Madrid, Madrid, Spain; ^2^Research Institute Hospital 12 Octubre (I+12), Madrid, Spain; ^3^Immunology Department, University Hospital 12 de Octubre, Madrid, Spain; ^4^Pediatric Nutrition, Pediatrics, University Hospital 12 de Octubre, Complutense University of Madrid, Madrid, Spain; ^5^School of Medicine, Complutense University of Madrid, Madrid, Spain; ^6^Immunology Department, University San Pablo CEU, Madrid, Spain; ^7^Pathology Department, University Hospital 12 de Octubre, Madrid, Spain; ^8^Pediatric Rheumatology Unit, Department of Pediatrics, University Hospital 12 de Octubre, Complutense University of Madrid, Madrid, Spain; ^9^Pediatric Hematology and Oncology, Hospital Virgen de la Salud, Toledo, Spain

**Keywords:** factor of kappa light polypeptide gene enhancer in B-cells 1, *NFKB1* gene, primary immunodeficiency, common variable immunodeficiency, mendelian susceptibility to mycobacterial disease, *Mycobacterium genavense*, submucosal lymphocytic plexitis, Langerhans cell histiocytosis

## Abstract

**Background:** NF-κB1 is a master regulator of both acquired and innate responses. *NFKB1* loss-of-function mutations elicit a wide clinical phenotype with asymptomatic individuals at one end of the spectrum and patients with common variable immunodeficiency, combined immunodeficiency or autoinflammation at the other. Impairment of acquired and innate immunity and disseminated *Mycobacterium genavense* infection expands the clinical and immunological phenotype of NF-κB1 deficiency.

**Objective:** Functional and molecular characterization of a patient with a novel phenotype of NF-κB1 deficiency.

**Methods:** Circulating T, B, dendritic cell subsets and innate or unconventional T-cells were quantified. The cytokine production in stimulated whole blood samples was assessed and molecular characterization by next generation sequencing and gene expression assays were also performed.

**Results:** We report a patient presenting with features of combined immunodeficiency (CID) and disseminated *Mycobacterium genavense* infection. Sequencing of genomic DNA identified a novel synonymous mutation (c.705G > A) in *NFKB1* gene which resulted in exon 8 skipping and haploinsufficiency of the NF-κB1 subunit p50. The susceptibility to atypical mycobacterial infection has not been previously reported and may be the result of a dendritic cell deficiency. A selective deficiency of circulating follicular helper T (cTFH) cells responsible for mediating the differentiation of naive B cells into memory and plasma cells was also present in the patient. It could affect the maturation of innate or unconventional T cells where NF-κB1 could also be involved.

**Conclusion:** These findings showed that the role of NF-κB1 in humans could be critical for the development of acquired and innate immunity and further highlights the role of human T cells in anti-mycobacterial immunity.

## Key Points

- The clinical spectrum of NF-κB1 deficiency in humans is expanding and includes increased susceptibility to atypical mycobacterial infection.
- Pleiotropic immune defects can be identified in conventional and unconventional T compartment and dendritic cells, recapitulating the phenotype of NF-κB1 deficiency.

## Introduction

The nuclear factor kappa light-chain enhancer of activated B-cells (NF-κB) is a pleiotropic transcription factor present in almost all cell types and represents the endpoint of a series of signal transduction events initiated by a vast array of stimuli related to many biological processes such as inflammation, apoptosis, differentiation, cell growth, tumorigenesis, and immunity (1). Mutations in several genes encoding molecules of the NF-κB pathway have been associated with primary immunodeficiencies (2) including new combined and antibody defects such as CARD-BCL10-MALT1 (3) and NF-κB2 (4), respectively.

Heterozygous mutations of *NFKB1* gene lead to haploinsufficiency of NF-κB1. It was initially described in patients with common variable immunodeficiency (CVID) (5). The presentation of NF-κB1 deficiency has since expanded to include more diverse immunologic phenotypes ranging from combined immunodeficiency(CID) (6, 7) to autoinflammatory disease (8, 9), broadening the clinical phenotype.

In this report, we present the clinical and immunological phenotype of a family with a novel synonymous mutation in *NFKB1* gene that affected the canonical splicing of the gene resulting in skipping of exon 8 and reduced expression of the NF-κB1 p105 and p50.

The patient suffered from disseminated *Mycobacterium genavense* infection, due to a CID that affected his acquired and innate immunity. *Mycobacterium genavense* is a relatively new species of non-tuberculous mycobacterium reported to cause disseminated infections in primary and secondary immunodeficiencies (i.e., AIDS). We also studied two asymptomatic mutation-carrying relatives without the clinical phenotype.

## Methods

### Immunophenotyping and Functional Assays

Immunophenotyping was performed on peripheral blood for the identification of T, B, NK, and dendritic cells (DCs). Conjugated anti-human monoclonal antibodies are listed in Supplementary Table 1. Flow cytometry data were collected using a Beckman Coulter Navios cytometer and analyzed with Kaluza 1.5a software (Beckman Coulter, Indianapolis IN, US).

Cytokines in stimulated whole blood were measured with ProcartaPlex™ 25-plex Immunoassay (Thermo Fisher) using Luminex®. Standard curves were constructed to interpolate analytes using ProcartaPlex Analyst version 1.0. The mean of technical duplicates was recorded.

### NGS and Sanger Sequencing

Genomic DNA was extracted from EDTA blood samples using the QIAmp DNA Mini Kit (Qiagen, Hilden, Germany). NGS were done by targeted gene sequencing with an in-house designed panel of 192 genes involved in primary immunodeficiency (PID) (Ampliseq, Life Technologies) (Supplementary Table 2) and by whole exome sequencing (WES) in the trio family. WES was based on an Illumina HiSeq2000 sequencing platform and an Agilent's SureSelect Target Enrichment System for 51 Mb. The reads were aligned against the human reference genome hg38 using the Burrows-Wheeler Alignment tool (BWA) (10). After reads mapping, low-quality reads and PCR duplicates were removed and with Picard Tools. For the variant calling process, different algorithms were applied, including VarScan (11) and the Genome Analysis Toolkit (GATK) (12). Python scripts were developed to combine variants. Variants annotation was based on Ensembl and NCBI databases.

Variants were filtered according to an autosomal dominant inheritance model. It is shown a schematic overview of the strategy used to filter variants through WES in order to identify potentially causative mutations (see Supplementary Figure 1).

*NFKB1* synonymous variant was confirmed by Sanger sequencing. For DNA amplification, reactions were carried out in 100 μL containing 5 U Taq DNA Polymerase (Perkin Elmer), 200 μM dNTPs, 0.5 μM of each primer and 1 μg of genomic DNA. Primers used for amplification of exon 8 of *NFKB1* gene were: gNFKB1 intron7 Forward 5′: TTGGGCTTTATAAAAGCATGG, and gNFKB1 intron8 Reverse 5′: GGCAGGGCTGGAAGTCTATT. PCR conditions were as follows: one cycle of 5 min at 95°C and 35 cycles of PCR (15 s at 95°C, 30 s at 58°C, and 40 s at 72°C), followed by 10 min at 72°C for the final elongation. RNA was extracted from peripheral blood lymphocytes from the patient and their family members by using RNeasy plus mini kit (Quiagen, Madrid, Spain). Reverse transcription was done on 0.5 μg of cytoplasmatic RNA, using a one-step RT-PCR method (Invitrogene), by using specific primers for the reaction that cover from exon 7 to exon 9 of *NFKB1* gene. The primers used were: NFKB1_mRNA ex7Forward 5′-TTGAAACACTGGAAGCACGA and NFKB1_mRNA ex9Reverse 5′-ATTTCCTCCCCTCCAGTCAC. RT-PCR conditions were as follows: one cycle of RT (20 min at 50°C followed by 5 min at 95°C) and 35 cycles of PCR (15 s at 95°C, 30 s at 57°C, and 50 s at 72°C), followed by 10 min at 72°C for the final elongation. PCR products were screened by direct cycle sequencing. Double-strand DNA templates were sequenced using the dideoxy chain-terminator method of Sanger, with Applied Biosystems DyeDeoxy terminators.

### Gene Expression Assays

Gene expression was analyzed by real-time PCR using a TaqMan Fast Universal PCR Master Mix and Taqman probes (NFKB1: Hs00765730-m1) (Thermo Fisher) in accordance with the manufacturer's instruction. GADPH was used as the endogenous control, and the level of expression of *NFKB1* of the patient, father and sibling were quantitatively measured in duplicates relative to that in two different healthy donors. Western blotting was done according to methods described (5).

All experimental work was performed after written informed consent for publication of clinical and immunological information of the patient was provided from his parents and all adult participants. All human subject samples were consented under protocols approved by the Institutional Review Board (IRB) of our Institution. The study was approved by the IRB at Hospital 12 de Octubre. The study fulfilled the IRB standards for ethical conduct of research with human subjects.

### Statistical Analysis

The phenotypic studies were performed on the patient, brother and healthy donors at the age of 6.8 ± 0.4, 10.75 ± 0.35, and 4.8 ± 1.6 years (mean ± SD), respectively. Significant differences were determined by using non-paired Student *t*-test (Prism; GraphPad software, La Jolla, Calif). A *p*-value of < ^*^0.05, ^**^0.01, and ^***^0.001 were considered significant.

## Results

### Case Presentation

**Figure 1:**
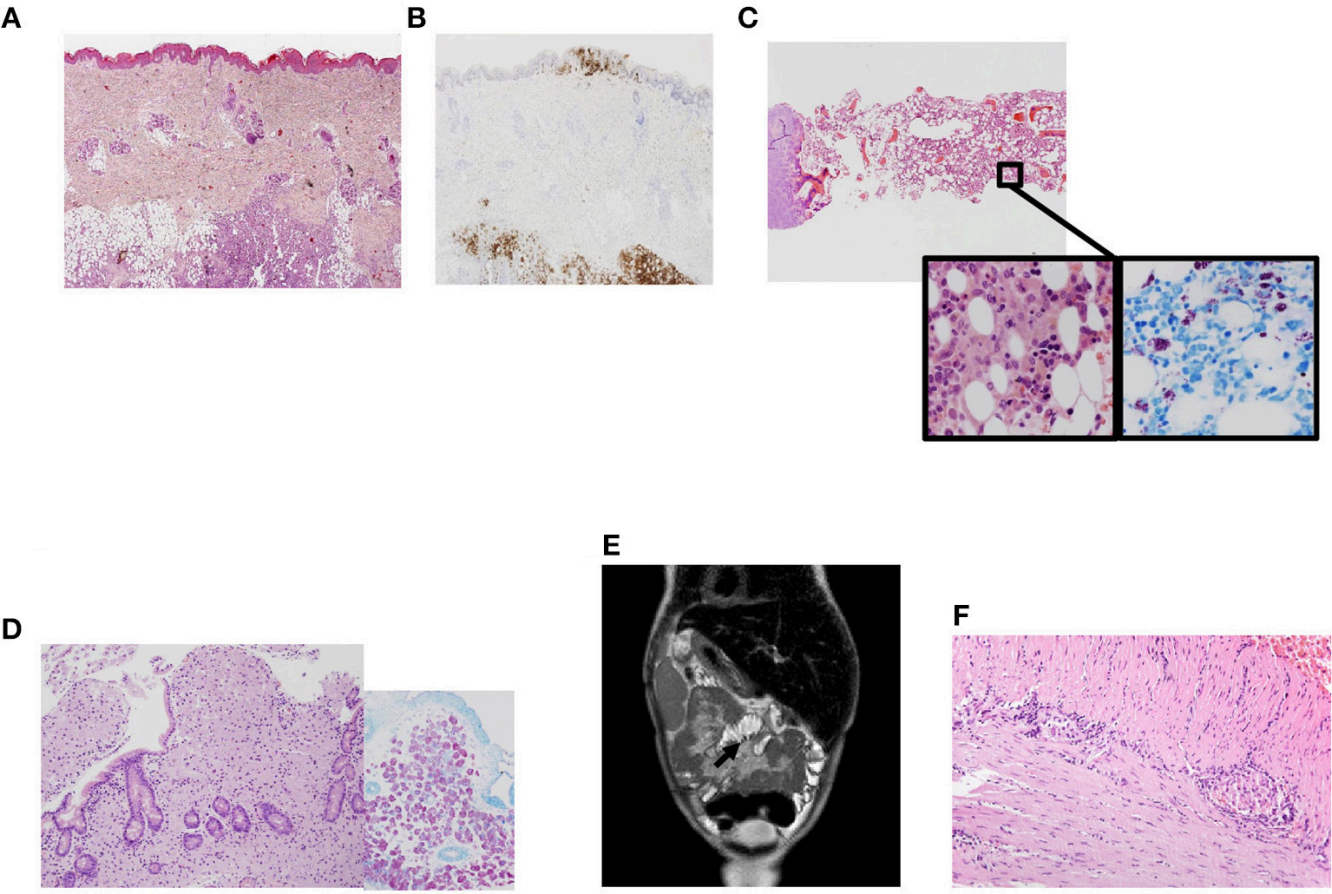
Clinical phenotype in a patient with NF-κB1 deficiency. **(A)** Skin biopsy showing a dense cellular infiltrate by Langerhans cells. Magnification 200×. Hematoxilin-eosin; **(B)** Skin biopsy with Langerhans cells infiltrating epidermis and subcutaneous tissues, positive to CD1a. Magnification 200×. Immunohistochemistry CD1a; **(C)** Panoramic view of bone marrow with many histiocytes with granular cytoplasm. Magnification 40×. Inside images: high power view of histiocytes between hematopoietic cells. They were intensely positive with acid-alcohol techniques (Ziehl Nielsen). Magnification 400×. Hematoxilin-eosin and Ziehl Nielsen; **(D)** Panoramic view of duodenal biopsy with villi shortened and lamina propria expanded by many granular histiocytes that stained positive with Ziehl Nielsen to detect Acid-resistant bacilli (inside). Magnification 200×. Hematoxilin-eosin. Inside Ziehl-Nielsen. Magnification 200× **(E)** Post-contrast coronal T2-weighted MRI of the abdomen showing mesentery enhancement. **(F)** Small bowel biopsy showing myenteric plexus with lymphoplasmocitoid inflammatory cells. Magnification 200×.

A 7-years-old male born to non-consanguineous Caucasian parents presented to our center at the age of 8 months with cutaneous lesions on his trunk. Skin biopsy revealed Langerhans cell histiocytosis (LCH) (Figures 1A,B). As the disease progressed (cutaneous and mucosal disease), systemic steroids were added achieving partial remission 3 months later. Shortly thereafter he developed worsening anemia, fever, marked hepatosplenomegaly, and oral ulcers. Radiographic skeletal survey imaging revealed lytic lesions in skull and tibia indicating disease progression. Bone marrow aspirate and trephine biopsy did not show infiltration. At this time, he was 18-months-old and was treated according to protocol LCH-IV. During the continuation phase he received clofarabine due to refractory disease (13).

At the age of 3 years LCH was in remission and methotrexate and mercaptopurine were started as maintenance therapy. One month after starting treatment, he developed febrile neutropenia, abdominal pain and night sweats. Biopsies were obtained from bone marrow and gut detecting acid-alcohol resistant bacilli identified as *Mycobacterium genavense* by PCR techniques (Figures 1C,D). The patient required four intravenous antimycobacterial drugs (rifampin, ethambutol, clarithromycin, and levofloxacin) at standard doses and improved clinically. Follow-up biopsies taken from both gut and bone marrow 1 year after starting specific therapy demonstrated clearing of non-tuberculous mycobacterial bacilli. The patient continued complaining of chronic abdominal pain which was attributed to post-chemotherapy enteritis. Due to the persistence of the pain an MRI was obtained (Figure 1E) revealing sclerosing mesenteritis. Systemic corticosteroids were then added. The patient developed severe protein-losing enteropathy with malabsorption, becoming steroid-dependent and requiring long-term parenteral nutrition. A new gut biopsy revealed chronic lymphocytic plexitis (Figure 1F). He had prolonged shedding after viral infections (RSV and norovirus, both requiring specific treatment with ribavirin in both cases).

During the last 2 years the patient has been asymptomatic and free of infections. Anti-mycobacterial treatment was withdrawn 1 year ago without relapse. Currently, he is receiving oral clarithromycin as secondary prophylaxis.

### Immunologic and Genetic Profile of a Family With NF-κB1 Deficiency

The clinical and immunological phenotype of a family with a novel variant leading to splicing defect in *NFKB1* gene were studied. The index patient suffered disseminated *Mycobacterium genavense* infection due to a CID. The profile of the relatives and proband are described in Table 1. None of them had been vaccinated with BCG.

**Table 1:** Immunologic features of the family.

**Parameter**	**Ref values (3–10 y.o.)**	**Ref values (adults)**	**Patient 3 y.o**.	**Sibling 9 y.o**.	**Father adult**
Lymphocyte (n°/μL)	2,500–6,000	1,200–3,000	1,737	3,406	1,993
**T CELLS**					
CD3^+^n°/mL (%)	1,400–4,300(52–88)	850–2,250(62–81)	1,541(89)	2,398(70)	1,321 (66)
CD3^+^ HLA-DR^+^ (%)	0–10	NA	31	14	NA
CD3^+^ TCRαβ (%)	85–99	NA	98	94	NA
CD3^+^ TCRαβ + DNT (%)	0–2.5	NA	0.2	1.1	NA
CD3^+^ TCRγδ (%)	2–15	NA	1	5	NA
CD4^+^ n°/μL (%)	800–2,500(33–55)	500–1,450(32–59)	293(17)	1,124(33)	817(41)
CD4^+^CD45RA^+^CCR7^+^ (Naïve) (%)	42–82	20–62	18.8	60.9	30.8
CD4^+^CD45RA^−^CCR7^+^ (CM) (%)	15–30	15–35	6.2	20.5	31.9
CD4^+^CD45RA^−^CCR7^−^ (EM) (%)	8–30	20–40	74,3	17.6	35.5
CD4^+^CD45RA^+^CCR7^−^ (E) (%)	0.4–4	0.4–5	0.7	0.99	1.9
CD4^+^CD45RA^+^CD31^+^ (%)	44–60	NA	10	45	NA
CD8^+^ n°/μL (%)	400–1,400(17–34)	160–950(15–36)	1,282(74)	1,192(35)	478(24)
CD8^+^CD45RA^+^CCR7^+^ (Naïve) (%)	30–80	10–50	2.3	53.1	23.5
CD8^+^CD45RA^−^CCR7^+^ (CM) (%)	3–28	5–20	1.3	4.9	7.9
CD8^+^CD45RA^−^CCR7^−^ (EM) (%)	17–35	10–40	91.1	29.5	37.4
CD8^+^CD45RA^+^CCR7^−^ (TEMRA) (%)	2–15	5–35	5.3	12.5	31.1
TRECs (n° Copies/μg DNA)	>10	NA	5.3	32	NA
**T CELLPROLIFERATION**					
Unstimulated	0–1,000	NA	878	356	NA
PHA (c.p.m.)	>30,000	NA	940	31,187	NA
Anti-CD3 (c.p.m.)	>10,000	NA	1,098	10,494	NA
Anti-CD3^+^Anti-CD28 (c.p.m.)	>30,000	NA	3,146	32,235	NA
PMA + Ionomicin (c.p.m.)	>35,000	NA	7,662	39,576	NA
**NK CELLS**					
CD56^+^CD3^−^ n°/μL (%)	100–650(2–20)	60–450(4–22)	87(5)	770(22.6)	419(21)
**B CELLS**					
CD19^+^ n°/μL (%)	400–1,500(9–28)	100–500(8–20)	52(3)	225(6.6)	239(12)
CD19^+^CD27^+^ (%)	7–19	8–50	4.5	35.7	24
CD19^+^IgD^+^CD27^−^ (%Naïve)	75–89	59–88	95	59.4	75.3
CD19^+^IgD^+^CD27^+^ (%MZ)	2.6–7.1	3–12	0.9	18.3	13.8
CD19^+^IgD^−^CD27^+^ (%SW)	4.5–20	10–40	2.3	17.4	10.2
CD19^+^CD38hiIgM^+^ (%Transitional)	3–10	3–10	50.3	8.8	6.6
Plasmablasts	0.5–5	0.6–6	0.4	4.2	1.3
KRECs (n° Copies/μg DNA)	>10	NA	5.9	14	NA
**SERUM IMMUNOGLOBULINS (MG/DL)**					
IgG (mg/dL)	600–1,230	700–1,600	958	967	1,310
IgA (mg/dL)	30–200	70–400	74	219	404
IgM (mg/dL)	50–200	40–230	49	102	198
**SPECIFIC ANTIBODIES**					
IgG vs. Pneumococcus (mg/dL)	>5.4	NA	0.6	NA	NA
IgG2 vs. Pneumococcus (mg/dL)	>2.4	NA	0.1	NA	NA
IgG vs. Tetanus toxoid (IU/mL)	>0.1	NA	0.86	1.9	NA
IgG a-HBsAg	>10	NA	Negative	NA	NA

*DNT: CD3^+^TCRαβ^+^CD4^−^CD8^−^ Double negative T-cell. HBsAg, Hepatitis B surface antigen*.

**Figure 2:**
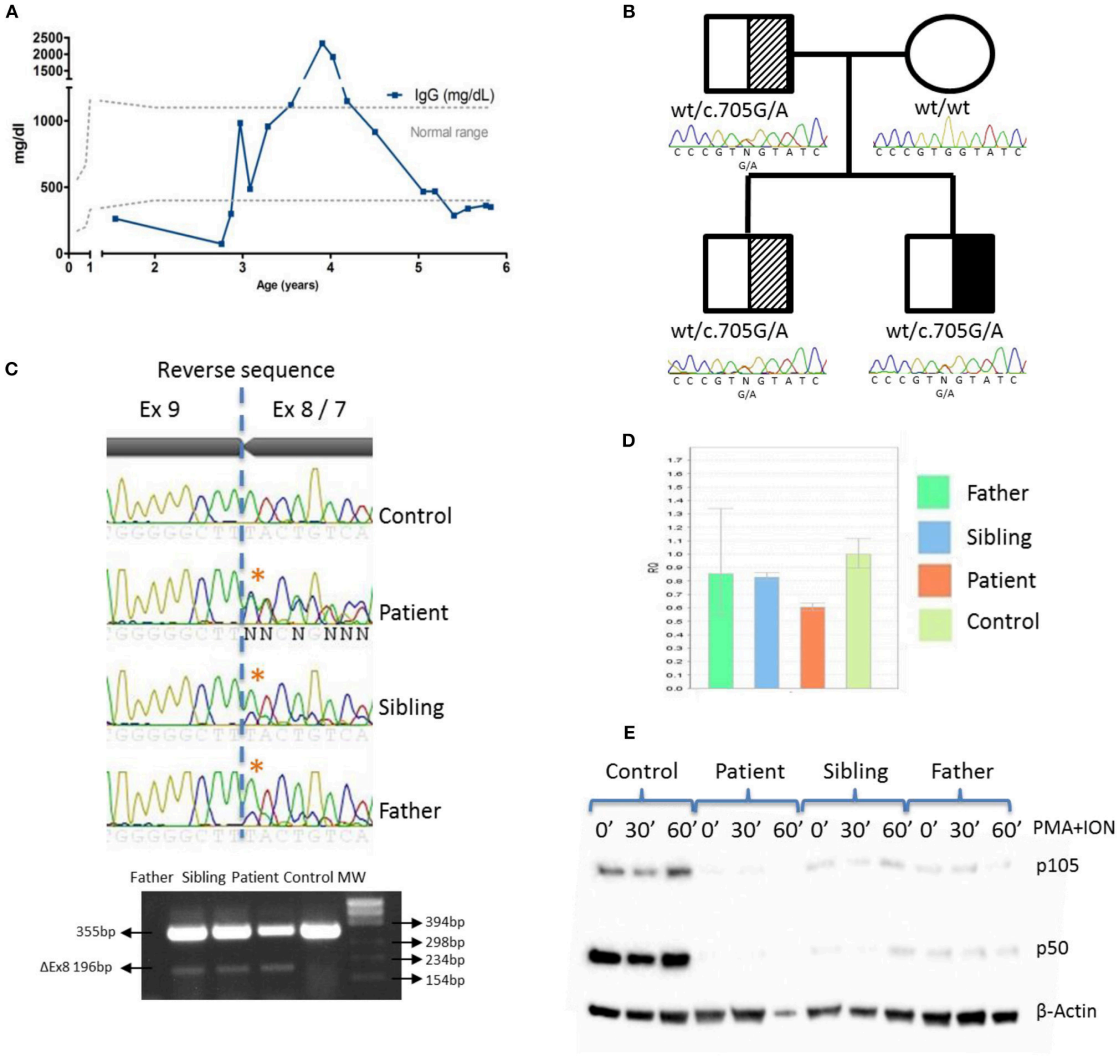
IgG levels and genetic and functional evaluation in a patient with NF-κB1 deficiency. **(A)** Serum IgG concentrations over time. **(B)** Pedigree and genomic sequencing of c.705G > A *NFKB1* gene variant. Black represents the affected individual (patient). Shaded represents asymptomatic carriers; **(C)** Sanger sequencing and RT-PCR amplification of *NFKB1* mRNA showing exon 8 skipping; **(D)** *NFKB1* expression from patient, father and sibling relative to controls by qRT-PCR; **(E)** The mutation led to reduced expression of p105 and p50 upon stimulation with PMA + ION in patient, father and sibling by western blotting.

Immunophenotyping of the patient showed CD4, B, and NK cell lymphopenia at the age of 3 years. Serum immunoglobulins were normal until he was five and a-half-years-old, when decreasing IgG levels led to suspicion of a primary immunodeficiency (Table 1; Figure 2A).

A targeted-NGS panel of 192 PID related genes (Supplementary Table 2) revealed a heterozygous nucleotide substitution in exon 8 of *NFKB1* (c.705G > A) gene, not found in gnomAD or 1,000 genomes databases, that was predicted to be silent (p.Val235Val). No additional putative disease-causing mutations were identified by whole exome sequencing (WES) (Supplemental Figure 1). Sanger sequencing confirmed that the patient inherited the variant in an autosomal dominant fashion with variable penetrance from his father who is healthy. The patient has an older healthy sibling, which also carries the *NFKB1* mutation (Figure 2B). We also evaluated the potential functional impact of this mutation by assessing its influence on gene splicing with several computational tools (data not shown) (14), indicating that a cryptic donor splice site just upstream of this mutation could be activated, which would result in exon 8 skipping and assigned this variant as pathogenic. To verify this prediction, we sequenced a *NFKB1* cDNA fragment from exons 7 to 9, confirming an additional shorter product (Δ159bp) that corresponded to exon 8 skipping (Figure 2C). However, we cannot rule out that a deep intronic mutation provokes exon 8 skipping. The skipping of exon 8 resulting in an in-frame 53aa deletion (p.Asp191_Lys244delinsGlu) and p50 haploinsufficiency (5) has been previously reported.

*NFKB1* gene expression assays showed that the symptomatic patient presented less WT transcripts than the other mutation positive relatives (Figure 2D). The mutated protein p105ΔEx8 led to rapid degradation and to haploinsufficiency of the p50 protein (Figure 2E). These data confirmed that a heterozygous germline synonymous mutation (g.Chr4:103.500.171G > A; p.Asp191_Lys244delinsGlu) in *NFKB1* may be responsible for the novel form of CID in this patient. Similar to other gene defects with an impact on immune system control, *NFKB1* mutations lack complete penetrance (15).

### Impact of NFKB1 Mutation in Acquired Immunity

**Figure 3:**
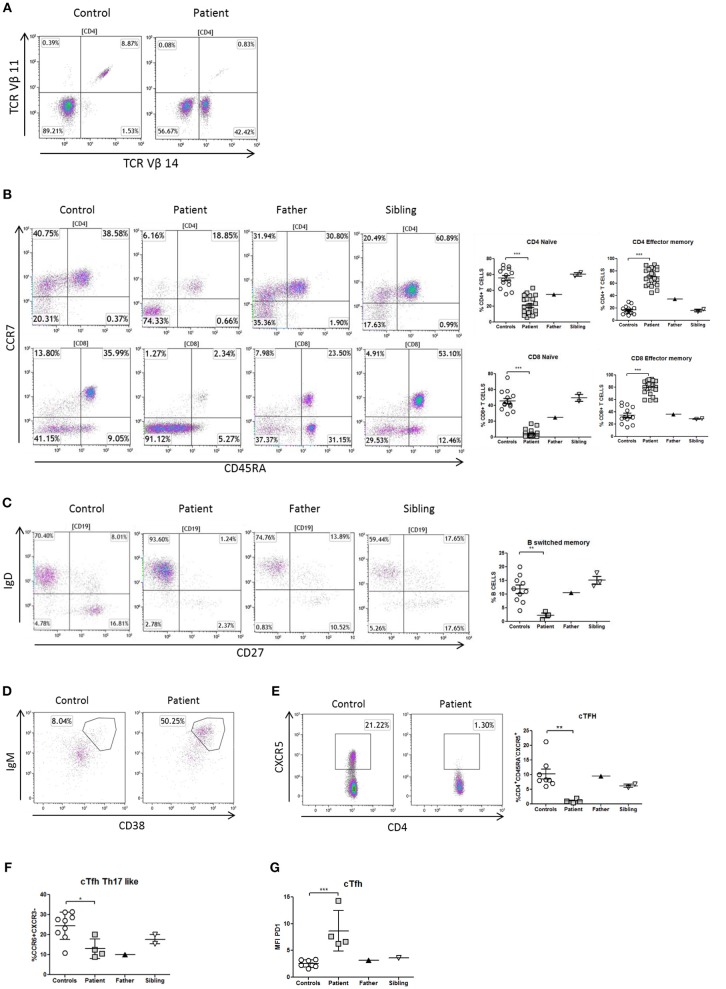
Immunophenotype of T- and B-cells in a patient with NF-κB1 deficiency. **(A)** TCRVβ repertoire: the patient showed an expansion of the family CD3^+^TCRαβ^+^Vβ14^+^ compared to healthy control; **(B)** Decrease naïve (CCR7^+^CD45RA^+^) and high levels of T-effector memory phenotype (CCR7-CD45RA-) CD4 and CD8 T-cells in the patient; It is also shown the dot plot of the father and sibling **(C)** The patient showed reduced switched memory B-cells (CD19^+^CD27^+^IgD^−^) compared to father, sibling and healthy controls; **(D)** The patient showed expansion of immature transitional B cells (CD19^+^CD38^++^IgM^++^) and **(E)** deficiency of circulating follicular helper T (cTFH) cells (CD4^+^CXCR5^+^) compared to healthy control. **(F)** The patient showed reduced Th17-like, and **(G)** increased expression (MFI) of PD1^++^ in TFH-cells. The comparison in this figure was done with age matched healthy donors. Lines represent mean and bars represent the standard error of the mean. ^*^P < 0.05, ^**^P < 0.01, ^***^P < 0.001.

The immunophenotype of the patient showed defective thymopoiesis with reduced recent thymic emigrants and T-cell receptor rearrangement excision circles (TRECS), restricted T-cell repertoire, decreased naïve and increased effector phenotype in CD4 and CD8 T-cells, as well as an impaired lymphoproliferative response to mitogens (Table 1; Figures 3A,B). These findings could be related to a senescent T-cell phenotype. Absolute counts and proportion of B-cells were low, with decreased immunoglobulin serum concentrations and absent vaccine responses to pneumococcal polysaccharide as well as to hepatitis A and B antigens prior to immunoglobulin replacement therapy (Table 1 and Figure 2A). The patient had reduced switched memory B-cells and KRECS and expansion of transitional B cells (Figures 3C,D; Table 1). These findings were consistent with a CID phenotype where NF-κB1 haploinsufficiency could also be involved.

Differentiation of naïve CD4 T-cells into a specialized memory CD4 T-cells named circulating follicular helper T (cTFH) cells is essential to produce a subset with the most efficient helpers for B-cell differentiation. In this context and expanding the immunological phenotype of NF-κB1 deficiency, we have identified in this patient a cTFH deficiency with lacking CD4^+^CXCR5^+^ T-cells (Figure 3E). At the same time cTFH can be divided into different subpopulations according to CCR6 and CXCR3 expression of CD4 T-cells and cytokine production. The patient showed a low distribution of Th17-like memory subset (CD4^+^CXCR5^+^CD45RA^−^CCR6^+^CXCR3^−^) cells, which are key promoters of immunoglobulin secretion (Figure 3F). Moreover, cTFH from the patient had an increased PD1 expression in comparison to healthy donors (Figure 3G).

**Figure 4:**
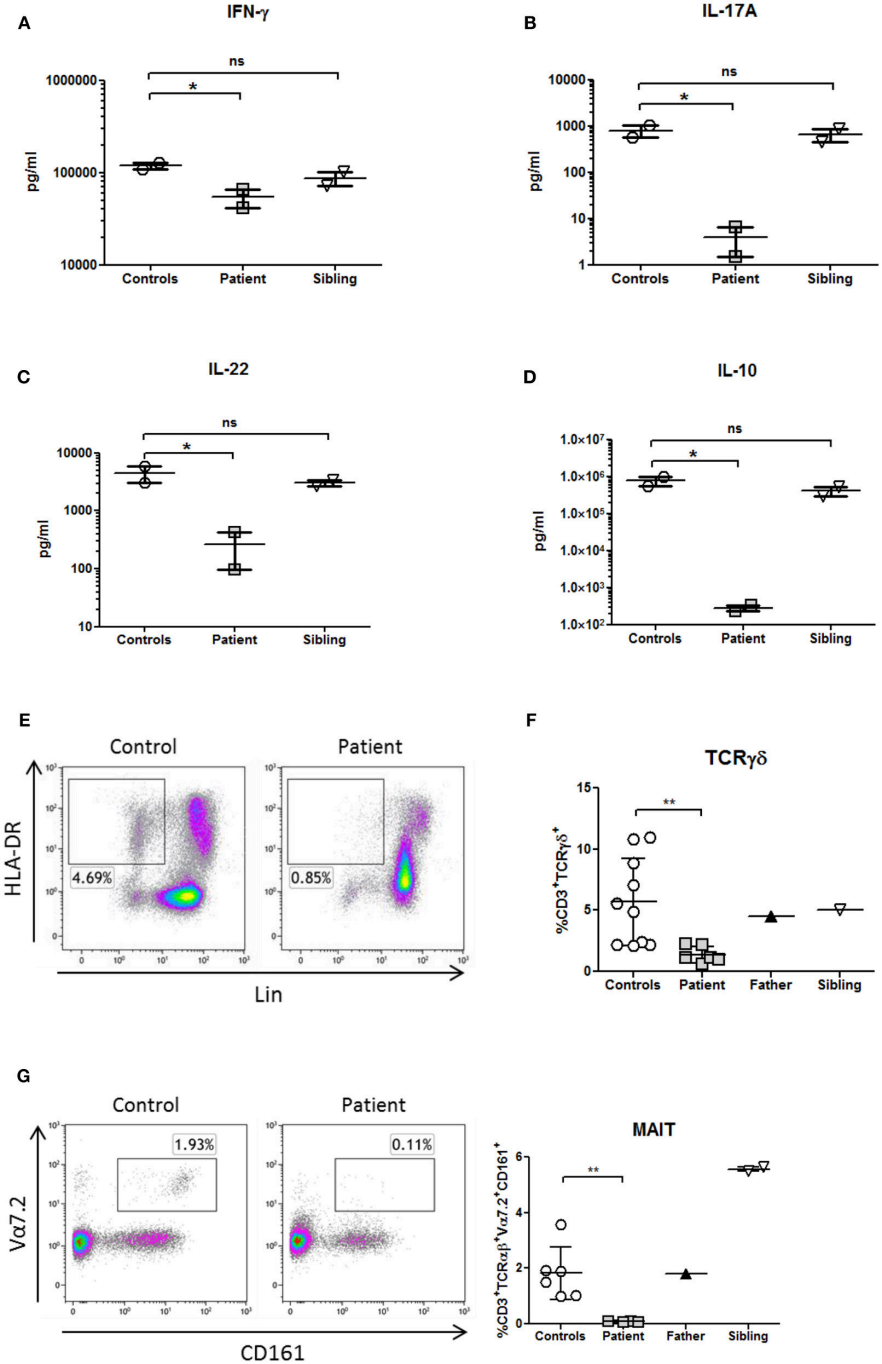
Cytokines profile and innate immunophenotyping in a patient with NF-κB1 deficiency. Cytokines were measured in supernatants from whole blood samples by Luminex and stimulated with PHA overnight (10 μg/mL; Merck) **(A)** IFNγ production (Th1 cytokine) **(B,C)**, IL-17A, and IL-22 production (Th17 cytokines) and **(D)** IL-10 production (B-cell helper/TFH cytokine). All samples were assayed in duplicate in two different experiments. **(E)** Deficiency of dendritic cells (DCs) (HLA-DR^+^Lin^−^), **(F)** CD3^+^TcRγδ^+^, and **(G)** mucosal-associated invariant T (MAIT) T-cells (CD3^+^TCRαβ^+^Vα7.2^+^CD161^+^) is shown in the patient. The comparison in this figure was done with age matched healthy donors. Lines represent mean and bars represent the standard error of the mean. ^*^P < 0.05, ^**^P < 0.01. Statistical comparisons were performed with unpaired Student *t*-tests, with significance defined as *P*-values ^*^P < 0.05, ^**^P < 0.01.

Whole blood assays showed that Th1 production (IFNγ), Th17 (IL-17A, IL-22) and B-cell helper/TFH (IL-10) cytokines were decreased in response to phytohemagglutinin (PHA) in comparison with healthy donors and healthy brother, according to the immunophenotype of the patient (Figures 4A–D).

### Impact of NFKB1 Mutation in Innate Immunity

Analysis of patient's PBMCs confirmed a severe deficiency of the non-lymphoid (CD3-CD19-CD56-) HLA-DR^+^ DCs (Figure 4E) with normal pDCs and mDCs distribution. However, IL-12p70 and IL-12p40 production in response to LPS was preserved (data not shown), probably due to the secretion of those cytokines by monocytes.

TCRγδ and MAIT cells are innate or “unconventional” T cells that recognize lipids, small-molecule metabolites, and specially modified peptides and respond killing target cells, releasing cytokines (IL-17, IL-22 and others) and activating and regulating other cells of the immune system (16). In this context, TCRγδ and MAIT cells from the patient were decreased in comparison to healthy controls (Figures 4F,G; Table 1). As previously shown, the production of IL-17A and IL-22 cytokines were also reduced in the patient in the context of a Th17 deficiency (Figures 4B,C).

## Discussion

*Mycobacterium genavense* is a ubiquitous non-tuberculous mycobacterium, first described as a human infection in the 1990s as a primary cause of fatal disseminated infection in patients with AIDS (17). *M. genavense* is also recognized as an opportunistic pathogen in patients without HIV who have secondary immunodeficiencies, including solid-organ transplantation, hematopoietic stem cell transplantation or immunosuppressive therapy (18). In this report, we describe a novel severe disseminated *Mycobacterium genavense* infection in a patient with NF-κB1 deficiency. LCH and the immunosuppressive treatments received by the patient initially led us to suspect a secondary immunodeficiency. However, the long-term CID finally made us consider a primary immunodeficiency. The two greatest decreases in naïve CD4^+^CCR7^+^CD45RA^+^ lymphocytes were secondary to chemotherapy at 3 years of age and due to protein-losing enteropathy at age 5 years, respectively (Supplementary Figure 2 and Supplementary Table 3). Previous reports of patients with the same molecular defect only showed a mild to moderate clinical and immunological impairment (19). The condition could partially resemble mendelian susceptibility to mycobacterial disease (MSMD), but mutations in MSMD genes and congenital defects of phagocyte number or function (20) were ruled out. It is well-known that a number of PIDs as well as the MSMD group share the susceptibility to mycobacterial disease due to lack of IFNγ production. Not surprisingly, our patient has decreased IFNγ production (21). Finally, NGS confirmed a NF-κB1 deficiency. It is likely that this immunophenotype was due to NF-κB1 deficiency in a patient with LCH and its sequelae. During the last 2 years the patient has been asymptomatic and free of infections. Anti-mycobacterial treatment was withdrawn 1 year ago without relapse. Despite these facts, the immunological phenotype of the patient was maintained over time (for full correlation between clinical and immunological phenotype, see Supplementary Table 3).

Several findings highlight that interactions between dendritic cells (DCs), CD4^+^ T-cells and B-cells are required for TFH formation (22). As in this case, the deficiency of DCs and the impairment of T- and B-compartment could produce a severe decrease of cTFH cells. These cells are responsible for mediating the differentiation of naïve B cells into memory cells and plasma cells, thereby providing effective humoral immunity against T-dependent antigens. For this reason, cTFH cells are used as a biomarker for dysfunctional humoral immunity (both immunodeficiency and autoimmunity). A wide spectrum of primary immunodeficiencies due to mutations in *CD40LG, ICOS, BTK*, and *STAT3* associate with TFH deficiency (23). A full comparison across PIDs with a putative TFH defect is summarized in Supplementary Table 4, where STAT3 LOF recapitulates the findings also found in our patient with NF-κB1 deficiency. NF-κB1 deficiency in humans could also have a critical role in TFH pathophysiology as it has been demonstrated in murine models (24).

It is well-known that patients with DC deficiencies are prone to mycobacterial infections due to pDCs (GATA2) or mDCs (IRF8) depletion, related to its ability to present mycobacterial lipid or viral antigens, respectively (25). DCs are specialized antigen-presenting cells (APCs), positioned within the immune system to bridge innate and adaptive immunity. In this case, it would be difficult to know whether DC deficiency had any role in the dissemination of *M. genavense* infection because IL-12 production was conserved in the patient; it could be hypothesized, however, that DC deficiency precluded the interplay with other immune cells, including B and NK lymphocytes and innate immunity (26). In this context, circulating MAIT cells correlate significantly with the number of switched memory B cells in CVID patients and require B cells for their development. In addition, MAIT cells are involved in antimycobacterial immunity although it is not known whether they possess B cell helper functions (27, 28). While the source of the defect in innate-like T cells in this case is unclear, it is possible that the markedly reduced numbers of antigen presenting cells (DCs and B cells) could produce an impaired development of MAIT and TCRγδ T-cells.

To summarize, NF-κB1 deficiency has a wide phenotypic variation ranging from asymptomatic individuals to severe autoinflammatory symptoms mimicking Behçet's disease and inflammatory gastrointestinal diseases (9). Thus, this synonymous *NFKB1* LOF heterozygous mutation produces a non-fully penetrant CID phenotype showing predisposition to non-tuberculous mycobacterial infections. This report illustrates the first case of a synonymous nucleotide substitution in the coding region of *NFKB1* gene that results in abnormal splicing. Next generation sequencing is an important part of the immunologist's toolbox and the need to evaluate the biologic relevance of synonymous variants will continue to increase (14, 29). Taken together, these data demonstrate a non-redundant role for NF-κB1 in regulating acquired and innate immunity in human immune cells. Development of non-tuberculous mycobacterial infection deserves proper immune assessment consisting of T- and B-cell compartments as well as DCs, MAIT and TCRγδ cells. The clinical phenotype of the patient to date has been dominated by non-tuberculous mycobacterial infection instead of CVID, as has been described in most cases of NF-κB1 deficiency.

## Author Contributions

RR-G performed the laboratory work for this study, computational predictions, and drafted the manuscript. ML-N and JB-E performed some of the laboratory work for this study. MG-D, JM-V, ND-P, JdI, and LG-G were responsible for the clinical management of the patients. OT and YR-G provided histopathological assessment and drafted the manuscript as EP-A did. LG-G and LA designed the research, collaborated in computational predictions, and drafted the manuscript. All authors approved the final version of this manuscript.

### Conflict of Interest Statement

The authors declare that the research was conducted in the absence of any commercial or financial relationships that could be construed as a potential conflict of interest.

## Abbreviations

CID: Combined immunodeficiency
NF-κB1: nuclear factor of kappa light polypeptide gene enhancer in B-cells 1
MSMD: Mendelian susceptibility to mycobacterial disease
IgG: immunoglobulin G
IgA: immunoglobulin A
IgM: immunoglobulin M
IgE: immunoglobulin E
PBMC: peripheral blood mononuclear cell
PID: primary immunodeficiency
TRECs: T-cell receptor rearrangement excision circles
KRECs: K-deleting recombination excision circles
MAIT cells: Mucosal associated invariant T cells.

## References

[1] Hayden MS Ghosh S. NF-κB in immunobiology. Cell Res. (2011) 21:223–44. 10.1038/cr.2011.13 21243012 PMC3193440

[2] Paciolla M Pescatore A Conte MI Esposito E Incoronato M Lioi MB. Rare mendelian primary immunodeficiency diseases associated with impaired nf-κb signaling. Genes Immun. (2015) 16:239–46. 10.1038/gene.2015.3 25764117 PMC4457537

[3] Pérez de Diego R Sánchez-Ramón S López-Collazo E Martínez-Barricarte R Cubillos-Zapata C Ferreira Cerdán A. Genetic errors of the human CARD-BCL10-MALT1 (CBM) complex: molecular, immunological, and clinical heterogeneity. J Allergy Clin Immunol. (2015) 136:1139–49. 10.1016/j.jaci.2015.06.031 26277595 PMC4894862

[4] Chen K Coonrod EM Kumánovics A Franks ZF Durtschi JD Margraf RL. Germline mutations in NFKB2 implicate the noncanonical NF-κB pathway in the pathogenesis of common variable immunodeficiency. Am J Hum Genet. (2013) 93:812–24. 10.1016/j.ajhg.2013.09.009 24140114 PMC3824125

[5] Fliegauf M Bryant VL Frede N Slade C Woon ST Lehnert K. Haploinsufficiency of the NF-κB1 subunit p50 in common variable immunodeficiency. Am J Hum Genet. (2015) 97:389–403. 10.1016/j.ajhg.2015.07.008 26279205 PMC4564940

[6] Boztug H Hirschmugl T Holter W Lakatos K Kager L Trapin D. NF-κB1 haploinsufficiency causing immunodeficiency and EBV-driven lymphoproliferation. J Clin Immunol. (2016) 36:533–40. 10.1007/s10875-016-0306-1 27338827 PMC4940442

[7] Ameratunga R Ahn Y Jordan A Lehnert K Brothers S Woon ST. Keeping it in the family: the case for considering late-onset combined immunodeficiency a subset of common variable immunodeficiency disorders. Expert Rev Clin Immunol. (2018) 14:549–56. 10.1080/1744666X.2018.1481750 29806948

[8] Schipp C Nabhani S Bienemann K Simanovsky N Kfir-Erenfeld S Assayag-Asherie N. Specific antibody deficiency and autoinflammatory disease extend the clinical and immunological spectrum of heterozygous NFKB1 loss-of-function mutations in humans. Haematologica (2016) 101:e392–6. 10.3324/haematol.2016.145136 27365489 PMC5046658

[9] Kaustio M Haapaniemi E Göös H Hautala T Park G Syrjänen J. Damaging heterozygous mutations in NFKB1 lead to diverse immunologic phenotypes. J Allergy Clin Immunol. (2016) 140:782–96. 10.1016/j.jaci.2016.10.054 28115215

[10] Li H Durbin R. Fast and accurate short read alignment with burrows-wheeler transform. Bioinformatics (2009) 25:1754–60. 10.1093/bioinformatics/btp324 19451168 PMC2705234

[11] Koboldt DC Chen K Wylie T Larson DE McLellan MD Mardis ER. VarScan: variant detection in massively parallel sequencing of individual and pooled samples. Bioinformatics (2009) 25:2283–5. 10.1093/bioinformatics/btp373 19542151 PMC2734323

[12] McKenna A Hanna M Banks E Sivachenko A Cibulskis K Kernytsky A. The genome analysis toolkit: a MapReduce framework for analyzing next-generation DNA sequencing data. Genome Res. (2010) 20:1297–303. 10.1101/gr.107524.110 20644199 PMC2928508

[13] Rodriguez-Galindo C Jeng M Khuu P McCarville MB Jeha S. Clofarabine in refractory Langerhans cell histiocytosis. Pediatr Blood Cancer (2008) 51:703–6. 10.1002/pbc.21668 18623218

[14] Gallego-Bustos F Gotea V Ramos-Amador JT Rodríguez-Pena R Gil-Herrera J Sastre A. A case of IL-7R deficiency caused by a novel synonymous mutation and implications for mutation screening in SCID diagnosis. Front Immunol. (2016) 7:443. 10.3389/fimmu.2016.00443 27833609 PMC5081475

[15] Rieux-Laucat F Casanova JL. Autoimmunity by haploinsufficiency. Science (2014) 345:1560–1. 10.1126/science.1260791 25258064

[16] Godfrey DI Uldrich AP McCluskey J Rossjohn J Moody DB. The burgeoning family of unconventional T cells. Nat Immunol. (2015) 16:1114–23. 10.1038/ni.3298 26482978

[17] Bessesen MT Shlay J Stone-Venohr B Cohn DL Reves RR. Disseminated *Mycobacterium genavense* infection: clinical and microbiological features and response to therapy. AIDS (1993) 7:1357–61. 10.1097/00002030-199310000-00009 8267909

[18] Hoefsloot W van Ingen J Peters EJ Magis-Escurra C Dekhuijzen PN Boeree MJ. *Mycobacterium genavense* in the Netherlands: an opportunistic pathogen in HIV and non-HIV immunocompromised patients. An observational study in 14 cases. Clin Microbiol Infect. (2013) 19:432–7. 10.1111/j.1469-0691.2012.03817.x 22439918

[19] Nijenhuis T Klasen I Weemaes CM Preijers F de Vries E van der Meer JW. Common variable immunodeficiency (CVID) in a family: an autosomal dominant mode of inheritance. Neth J Med. (2001) 59:134–9. 10.1016/S0300-2977(01)00151-6 11583829

[20] Picard C Bobby Gaspar H Al-Herz W Bousfiha A Casanova JL Chatila T. International union of immunological societies: 2017 primary immunodeficiency diseases committee report on inborn errors of immunity. J Clin Immunol. (2018) 38:96–128. 10.1007/s10875-017-0464-9 29226302 PMC5742601

[21] Boisson-Dupuis S Bustamante J El-Baghdadi J Camcioglu Y Parvaneh N El Azbaoui S. Inherited and acquired immunodeficiencies underlying tuberculosis in childhood. Immunol Rev. (2015) 264:103–20. 10.1111/imr.12272 25703555 PMC4405179

[22] Ma CS Wong N Rao G Nguyen A Avery DT Payne K. Unique and shared signaling pathways cooperate to regulate the differentiation of human CD4^+^ T cells into distinct effector subsets. J Exp Med. (2016) 213:1589–608. 10.1084/jem.20151467 27401342 PMC4986526

[23] Ma CS Wong N Rao G Avery DT Torpy J Hambridge T. Monogenic mutations differentially affect the quantity and quality of T follicular helper cells in patients with human primary immunodeficiencies. J Allergy Clin Immunol. (2015) 136:993–1006.e1. 10.1016/j.jaci.2015.05.036 26162572 PMC5042203

[24] Hu H Wu X Jin W Chang M Cheng X Sun SC. Noncanonical NF-kappaB regulates inducible costimulator (ICOS) ligand expression and T follicular helper cell development. Proc Natl Acad Sci USA. (2011) 108:12827–32. 10.1073/pnas.1105774108 21768353 PMC3150902

[25] Collin M Bigley V Haniffa M Hambleton S. Human dendritic cell deficiency: the missing ID? Nat Rev Immunol. (2011) 11:575–83. 10.1038/nri3046 21852794

[26] Halim TY Hwang YY Scanlon ST Zaghouani H Garbi N Fallon PG. Group 2 innate lymphoid cells license dendritic cells to potentiate memory TH2 cell responses. Nat Immunol. (2016) 17:57–64. 10.1038/ni.3294 26523868 PMC4685755

[27] Wong EB Ndung'u T Kasprowicz VO. The role of mucosal-associated invariant T cells in infectious diseases. Immunology (2017) 150:45–54. 10.1111/imm.12673 27633333 PMC5341498

[28] Arduini S Dunne J Conlon N Feighery C Doherty DG. Mucosal-associated invariant T cells are depleted and functionally altered in patients with common variable immunodeficiency. Clin Immunol. (2017) 176:23–30. 10.1016/j.clim.2016.12.002 28011187

[29] Platt CD Massaad MJ Cangemi B Schmidt B Aldhekri H Geha RS. Janus kinase 3 deficiency caused by a homozygous synonymous exonic mutation that creates a dominant splice site. J Allergy Clin Immunol. (2017) 140:268–71. 10.1016/j.jaci.2016.09.057 27956217 PMC5466846

