# Supplementary material for: Acquired and Innate Immunity Impairment and Severe Disseminated Mycobacterium genavense Infection in a Patient With a NF-κB1 Deficiency
Source: Front Immunol. 2019 Jan 29;9:3148. doi: 10.3389/fimmu.2018.03148 (PMC6362422; doi:10.3389/fimmu.2018.03148)
Supplement: Supplementary file 1 [file Data_Sheet_1.docx]

**Supplementary materials**

**Supplementary table 1.** Monoclonal antibodies.

| Antigen | Fluorochrome | Clone | Company |
| --- | --- | --- | --- |
| CCR7 | FITC | 150503 | BD Biosciences |
| CCR6 | PerCP-Cy5.5 | 11A9 | BD Biosciences |
| CD11c | APC | BU15 | Beckman Coulter |
| CD123 | PE | 9F5 | BD Biosciences |
| CD16 | FITC | NKP15 | BD Biosciences |
| CD161 | APC | 191B8 | Miltenyi Biotec |
| CD19 | PE-Cy7 | J4.119 | Beckman Coulter |
| CD21 | PE | B-ly4 | BD Biosciences |
| CD27 | APC-Cy7 | M-T271 | BD Biosciences |
| CD27 | PE | 1A4CD27 | Beckman Coulter |
| CD3 | Pacific Blue | UCHT1 | Beckman Coulter |
| CD3 | APC | UCHT1 | Beckman Coulter |
| CD3 | APC-AF750 | UCHT1 | Beckman Coulter |
| CD3 | PerCP-Cy5.5 | SK7 | BD Biosciences |
| CD38 | PerCP-Cy5.5 | HIT2 | BD Biosciences |
| CD4 | Krome Orange | 13B8.2 | Beckman Coulter |
| CD4 | APC-AF750 | 13B8.2 | Beckman Coulter |
| CD45 | APC | 2D1 | BD Biosciences |
| CD45 | Krome Orange | J33 | Beckman Coulter |
| CD45RA | APC-AF750 | 2H4LDH11LDB9 | Beckman Coulter |
| CD45RA | PE-Cy7 | L48 | BD Biosciences |
| CD56 | FITC | NCAM16.2 | BD Biosciences |
| CD56 | PE-Cy7 | NCAM16.2 | BD Biosciences |
| CD8 | APC | SK1 | BD Biosciences |
| CD8 | PerCP-Cy5.5 | SK1 | BD Biosciences |
| CD8 | FITC | SK1 | BD Biosciences |
| CXCR3 | PE | 1C6/CXCR3 | BD Biosciences |
| CXCR5 | APC | RF8B2 | BD Biosciences |
| HLA-DR | FITC | L243 | Beckman Coulter |
| HLA-DR | Pacific Blue | Immu-357 | BD Biosciences |
| IgD | FITC | IA6-2 | BD Biosciences |
| IgM | APC | G20-127 | BD Biosciences |
| PD-1 | PE-Cy7 | EH12.1 | BD Biosciences |
| TCRαβ | FITC | WT31 | BD Biosciences |
| TCRγδ | PE-Cy7 | IMMU510 | Beckman Coulter |
| TCRγδ | PE | B1 | BD Biosciences |
| TCRvβ 11 | PE | C21 | Beckman Coulter |
| TCRvα 24 | FITC | C15 | Beckman Coulter |
| TCRvα 7.2 | FITC | REA179 | Miltenyi Biotec |
| Lin3(CD3,CD14,CD19,CD20) | FITC | L27, MφP9, SJ25C1, SK7 | BD Biosciences |

**Supplementary table 2.** Gene-panel related to PID.

| ACT1 | ADA | AICDA | AIRE | AK2 |
| --- | --- | --- | --- | --- |
| AP3B1 | AP3D1 | ATM | BCL10 | BLNK |
| BTK | C3 | CARD11 | CARD9 | CASP10 |
| CASP8 | CD127 | CD19 | CD20 | CD21 |
| CD27 | CD3D | CD3E | CD3G | CD3Z |
| CD45 | CD79A | CD79B | CD81 | CD8A |
| CEBPE | CECR1 | NHEJ1 | CLEC7A | COPA |
| CORO1A | CTLA4 | CTPS1 | CTSC | CXCR4 |
| CYBA | CYBB | DCLRE1C | DKC1 | DNMT3B |
| DOCK2 | DOCK8 | ELANE | EVER1 | EVER2 |
| FADD | FCGR3A | FOXN1 | FOXP3 | G6PC3 |
| GATA2 | GFI1 | HAX1 | HOIL1 | ICOS |
| IFNGR1 | IFNGR2 | IGHM | IGLL1 | IKAROS |
| IKBA | IKBKB | IKBKG | IL10 | IL10RA |
| IL10RB | IL12B | IL12RB1 | IL12RB2 | IL17F |
| IL17RA | IL17RC | IL1RN | IL21 | IL21R |
| IL2RA | IL2RG | IL7 | IRAK4 | IRF3 |
| IRF7 | IRF8 | ISG15 | ITGB2 | ITK |
| JAGN1 | JAK3 | KIND3 | KRAS | LAMTOR2 |
| LCK | LIG4 | LPIN2 | LRBA | LYST |
| MAGT1 | MALT1 | MAP3K14 | MCM4 | MEFV |
| MHC2TA | MRE11 | MST1 | MVK | MYD88 |
| NCF1 | NCF2 | NFKB1 | NFKB2 | NHP2 |
| NLRC4 | NLRP12 | NLRP3 | NOD2 | NOP10 |
| NRAS | ORAI1 | p40phox | PGM3 | PIK3CD |
| PIK3R1 | PLCG2 | PMS2 | PNP | POLE1 |
| PRF1 | PRKCD | PSMB8 | PSTPIP1 | PTPN6 |
| RAB27A | RAG1 | RAG2 | RFX5 | RFXANK |
| RFXAP | RLTPR | RMRP | RNF168 | RORC |
| RTEL1 | SH2D1A | SMARCAL1 | SP110 | SPINK5 |
| STAT1 | STAT2 | STAT3 | STAT5B | STIM1 |
| STX11 | STXBP2 | TAP1 | TAP2 | TAPBP |
| TBK1 | TCF3 | TCN2 | TERC | TERT |
| TINF2 | TIRAP | TLR3 | TMEM173 | TNFRSF13B |
| TNFRSF13C | TNFRSF1A | TNFRSF5 | TNFRSF6 | TNFSF5 |
| TNFSF6 | TRAF3 | TRIF | TRNT1 | TTC7A |
| TWEAK | UNC119 | UNC13D | UNC93B1 | UNG |
| VPS45 | WAS | WIPF1 | XIAP | XRCC4 |
| ZAP70 | ZBTB24 |  |  |  |

**Supplementary table 3**. Clinical history and immunophenotype of the patient

| **Clinical history and Immunophenotype** | **Patient**  **8 months** | **Patient**  **2 y.o.** | **Patient**  **3 y.o.** | **Patient**  **4 y.o.** | **Patient**  **5 y.o.** | **Patient**  **6 y.o.** | **Patient**  **7 y.o.** |
| --- | --- | --- | --- | --- | --- | --- | --- |
| **Main Clinical events** | **Failure thriving** | **Failure thriving** | **Failure thriving** | **Failure thriving** | **Failure thriving** | **Failure thriving** | **Failure thriving** |
|  | **LCH** | **Flu B**  **RSV pneumonia** | **Active disseminated NTM infection**  **Abdominal pain** | **Abdominal pain** | **Chronic lymphocytic plexitis** | **Gut failure-TPN** | **Gut failure-TPN** |
|  |  |  | **Gut failure- TPN** | **Gut failure-TPN** | **Gut failure-TPN** |  |  |
| **Treatment** | **Topical steroids** | **LCH-IV* protocol**  **(Vinblastin, steroids, Clofarabine)**  **Abdominal pain** | **Anti NTM treatment**  **(iv)**  **Methylprednisolone** | **Anti NTM treatment**  **(iv)**  **Methylprednisolone** | **Anti NTM treatment**  **(iv/oral route)**  **Splenectomy**  **Methylprednisolone** | **Anti NTM prophylaxis** | **antiNTM prophylaxis**  **(ongoing)** |
| **Lymphocyte (nº/µL)** | 1866 | 4531 | 1737 | 2521 | 1000 | 4000 | 2269 |
| **T Cells** |  |  |  |  |  |  |  |
| **CD3+ nº/µL (%)** | 1455(78) | 3942(87) | 1541(89) | 2377(94) | 943(94) | 3755(94) | 1686(74) |
| **TCRαβ (%)** | **ND | ND | 98 | 97 | 99 | 99 | 99 |
| **TCRαβ DNT (%)** | ND | ND | 0.2 | 0.3 | 0.1 | 0.2 | 0.6 |
| **TCRγδ (%)** | ND | ND | 1 | 3 | 1 | 1 | 1 |
| **CD3+ HLA-DR+ (%)** | ND | ND | 31 | 16 | ND | 31 | 34 |
| **CD4+ nº/µL (%)** | 868(47) | 906(20) | 293(17) | 386(15) | 178(18) | 957(24) | 629(28) |
| **CD4+CD45RA+CCR7+ (Naïve) (%)** | ND | ND | 18.8 | 33.8 | 28 | 10.8 | 35.2 |
| **CD4+CD45RA-CCR7+ (CM) (%)** | ND | ND | 6.2 | 2 | 5.8 | 2.8 | 16.1 |
| **CD4+CD45RA-CCR7- (EM) (%)** | ND | ND | 74.3 | 60 | 65.5 | 83 | 44.5 |
| **CD4+CD45RA+CCR7- (E) (%)** | ND | ND | 0.7 | 3.9 | 0.3 | 3.3 | 4.2 |
| **CD8+ nº/µL (%)** | 582(31) | 2990(66) | 1282(74) | 997(71) | 769(77) | 2806(70) | 1053(46) |
| **CD8+CD45RA+CCR7+ (Naïve) (%)** | ND | ND | 2.3 | 0.7 | 3.7 | 3.4 | 16.6 |
| **CD8+CD45RA+CCR7- (CM) (%)** | ND | ND | 1.3 | 0.9 | 2.2 | 0.6 | 3.2 |
| **CD8+CD45RA-CCR7- (EM) (%)** | ND | ND | 91.1 | 86.2 | 88.8 | 79.6 | 71.8 |
| **CD4+CD45RA-CCR7+ (TEMRA) (%)** | ND | ND | 5.3 | 12.2 | 5.3 | 16 | 8.5 |
| **NK Cells** |  |  |  |  |  |  |  |
| **CD56+CD3- nº/µL (%)** | 187(10) | 544(12) | 87(5) | 85(6.1) | 14(1.3) | 80(2) | 107(4.7) |
| **B Cells** |  |  |  |  |  |  |  |
| **CD19+ nº/µL (%)** | 168(9) | 45(1) | 52(3) | 43(3.1) | 36(3.6) | 102(2.6) | 446(19.7) |
| **CD19+CD27+ (%)** | ND | ND | 4.5 | ND | 3.3 | 4.5 | 3.8 |
| **.0CD19+IgD+CD27- (%Naive)** | ND | ND | 95 | ND | 94.4 | 95 | 96 |
| **CD19+IgD+CD27+ (%MZ)** | ND | ND | 0.9 | ND | 1.9 | 0.9 | 1 |
| **CD19+IgD-CD27+ (%SW)** | ND | ND | 2.3 | ND | 2.33 | 3.6 | 2.1 |
| **CD19+CD38hiIgM+ (%Transitiomal)** | ND | ND | 50.3 | ND | 45.0 | 58.0 | 33 |

*The patient received chemotherapy according to LCH IV international protocol STRATUM I (weekly vinblastine 0.2 mg/Kg/dose for 12 weeks, and steroids 1.3 mg/Kg/day for 4 weeks, tapering over a period of 2 weeks, and then in three divided doses for 3 days every week, weeks 8-13).

As evaluation showed progressive disease, 6 cycles of clofarabine 30 mg/m2 every 4 weeks were administered, with good response.

After that, continuation therapy included methotrexate 20 mg/m2 weekly orally and 6-mercaptopurine 50 mg/m2 daily, combined with five-day prednisone 40 mg/m2/day and vinblastine 6 mg/m2 every 3 weeks. While he was receiving continuation treatment, he developed symptoms of mycobacterial infection.

**ND: not determined

**Supplementary table 4**.Comparison of the patient's TFH phenotype with other PIDs^23*^

| Gene defect | **TFH** | **CXCR3**  **TFH** | **CCR6**  **TFH** | **PD1** | **IFNγ** | **IL-17A** | **IL-10** | **IL-22** | **% Memory B cells** |
| --- | --- | --- | --- | --- | --- | --- | --- | --- | --- |
| NFKB1 PATIENT | **low** | **normal** | **low** | **high** | **low** | **low** | **low** | **low** | **low** |
| STAT3 LOF | low | high | low | high | low | low | low | low | low |
| IL21R | normal | normal | low | high | low | low | low | low | low |
| NEMO | Low | mild | low | Normal-high | low | low | normal | low | low |
| STAT1 GOF | normal | high | low | high | low | low | low | low | low |
| ICOS | Low | normal | low | Normal-high | normal | low | low | low | normal |
| BTK | Low | normal | normal | Normal-high | low | low | normal | normal | low |
| CD40LG | Low | mild | mild | normal-high | low | normal | high | mild | low |
| IL12RB1 LOF | mild | normal | mild | normal | low | low | normal | low | normal |
| IL10R | Low | normal | normal | normal | normal | high | high | high | low |
| STAT1 LOF | Normal | normal | normal | normal | normal | normal | normal | high | normal |

*The yellow highlight represents similarities among diverse primary immunodeficiencies and the patient.

**SUPPLEMENTARY FIGURE LEGENDS**

**SUPPLEMENTARY FIGURE 1.** Schematic of the multiple steps required for the identification and validation of variants.

**SUPPLEMENTARY FIGURE 2.** Clinical course of the patient showing naïve CD4+CCR7+CD45RA+ lymphocytes according to clinical events and treatments. The data above the dashed line represent normal values. TPN: total parenteral nutrition. LCH: Langerhans cell histiocytosis.
