# Supplementary figures and images for: Acquired and Innate Immunity Impairment and Severe Disseminated Mycobacterium genavense Infection in a Patient With a NF-κB1 Deficiency
Source: Front Immunol. 2019 Jan 29;9:3148. doi: 10.3389/fimmu.2018.03148 (PMC6362422; doi:10.3389/fimmu.2018.03148)

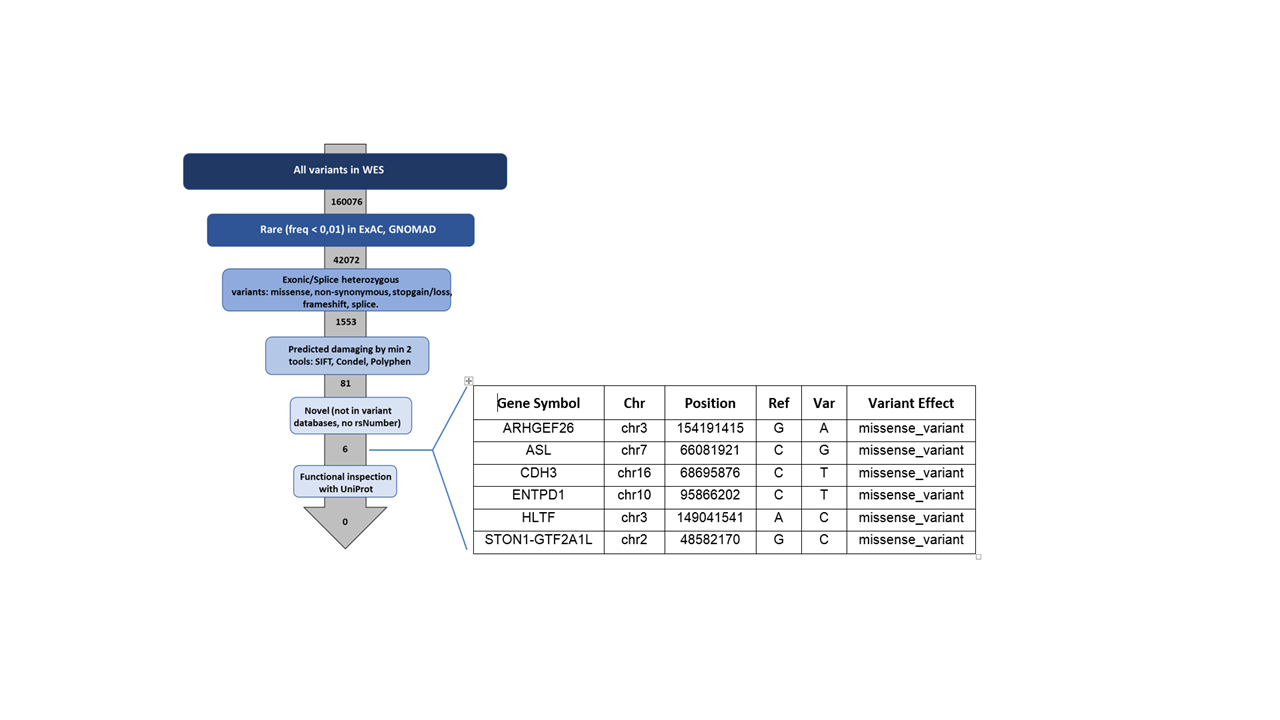

Supplement: Supplementary file 2 [file Image_1.TIF]

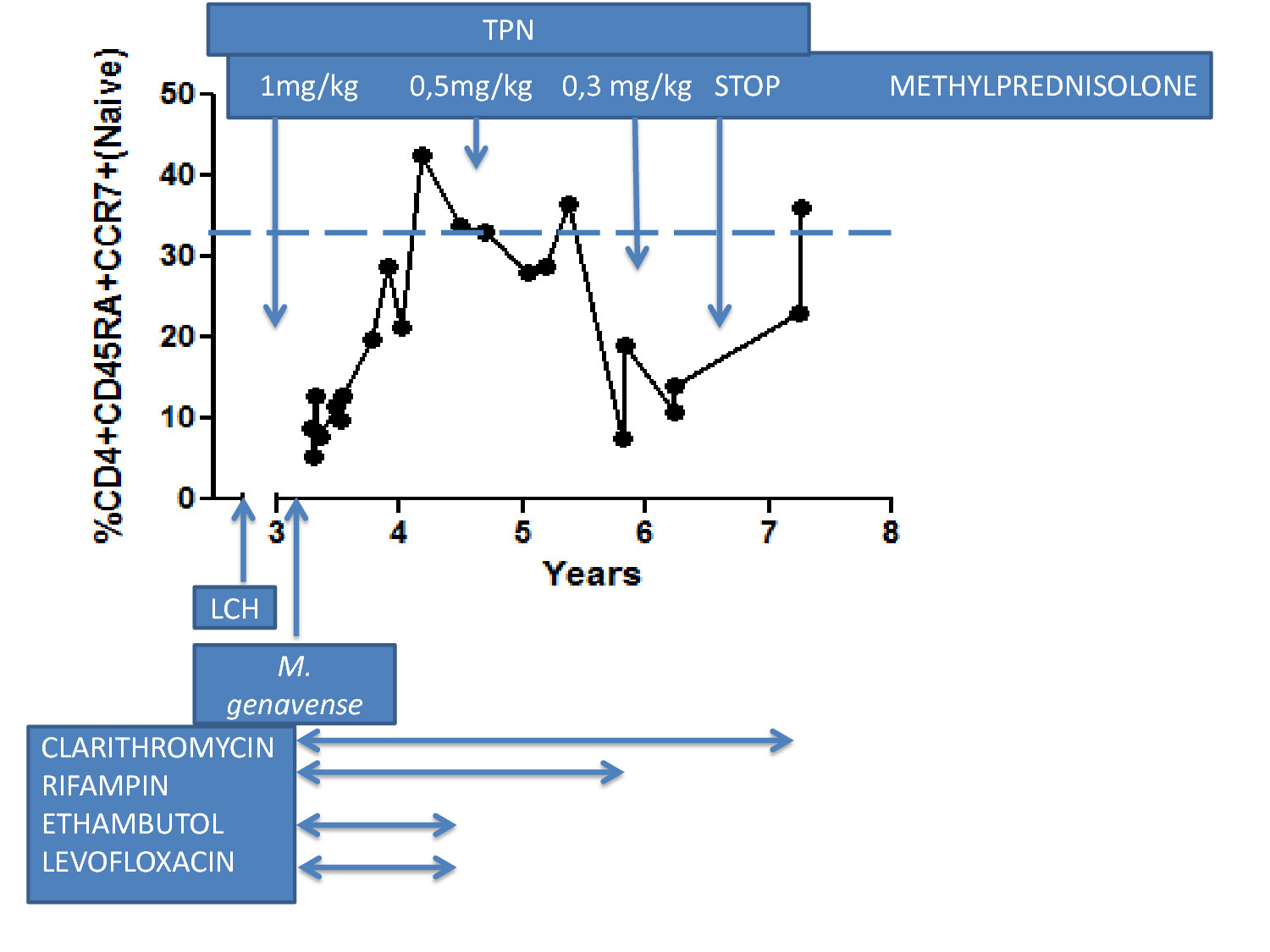

Supplement: Supplementary file 3 [file Image_2.JPEG]
